# Improving Access to Dementia Care for Native Older Adults Through Telehealth: A Community-Led Implementation

**DOI:** 10.1093/geroni/igaf122.1930

**Published:** 2025-12-31

**Authors:** Kala Cornelius, Jenna Woestman, Danielle Lennon, Celina Whitmore, Wesley Martin, Steve Barczi, Mary Wyman, Sarah Punshon

**Affiliations:** University of Wisconsin–Madison, Madison, Wisconsin, United States; Veterans Affairs, Madison, Wisconsin, United States; Veterans Affairs, Madison, Wisconsin, United States; Veterans Affairs, Madison, Wisconsin, United States; University of Wisconsin–Madison, Madison, Wisconsin, United States; Veterans Health Administration, Madison, Wisconsin, United States; UW–Madison and Veterans Affairs, Madison, Wisconsin, United States; William S. Middleton Memorial Veterans’ Hospital, Madison, Wisconsin, United States

## Abstract

The Native American population is rapidly aging and is disproportionately impacted by dementia. Native older adults and their family caregivers, especially those in rural areas, often face significant barriers to accessing dementia diagnosis, care, management and support services. Efforts to address these barriers through effective collaborations are critical to support aging in place. This presentation reports on a partnership between one Midwest Tribal Nation, Veterans Affairs, and the University of Wisconsin to tailor and implement comprehensive dementia care telehealth services. We report on our approach to developing collaborations with Tribal partners, delivery of geriatric education activities and telehealth implementation planning to date. We describe the implementation of dementia diagnosis and management services, delivered in the Tribal Health Clinic over telehealth by specialty geriatrics providers from the external healthcare systems and guided by a community engaged research approach. Infrastructure development and sustainment are facilitated through geriatrics-specific trainings for local Tribal Clinic staff and healthy aging education for community members. The authors report on development of the Tribal Telehealth Collaboration Roadmap (TTCR), a toolkit to guide other healthcare systems in dementia care telehealth delivery in the rural Tribal Clinic setting, based on our learned experience. The TTCR can support future dissemination efforts, minimize barriers to implementation, and increase access to comprehensive dementia care for Native older adults. Facilitators and barriers to implementation of a clinical program in the context of community engagement will be discussed.

